# Skeletal Muscle‐Specific Deletion of E3 Ligase *Asb2* Enhances Muscle Mass and Strength

**DOI:** 10.1002/jcsm.70007

**Published:** 2025-07-10

**Authors:** Hye Rim Jang, Shi‐Young Park, Yeonmi Lee, Dongjin Lee, Jongjun Lee, Yoonil Cho, Cheol Soo Choi, Hui‐Young Lee

**Affiliations:** ^1^ Laboratory of Mitochondria and Metabolic Diseases, Lee Gil Ya Cancer and Diabetes Institute Gachon University Incheon Republic of Korea; ^2^ Department of Health Sciences and Technology. GAIHST Gachon University Incheon Republic of Korea; ^3^ Korea Mouse Metabolic Phenotyping Center, Lee Gil Ya Cancer and Diabetes Institute Gachon University Incheon Republic of Korea; ^4^ Gachon Biomedical Convergence Institute Gachon University Gil Medical Center Incheon Republic of Korea; ^5^ Department of Molecular Medicine Gachon University School of Medicine Incheon Republic of Korea; ^6^ Department of Internal Medicine, Gil Medical Center Gachon University Incheon Republic of Korea

**Keywords:** ageing, *Asb2*, desmin, mitochondrial function, skeletal muscle mass

## Abstract

**Background:**

Maintaining skeletal muscle mass and strength is crucial to prevent sarcopenia during healthy ageing. Ankyrin repeat and suppressor of cytokine signalling box protein 2 (*Asb2*), an E3 ligase, has been implicated in regulating muscle mass; however, its roles on muscle strength remain unclear, with mixed findings from previous studies. Overexpression of *Asb2* decreases muscle mass, whereas its knockdown delays myoblast differentiation and reduces contractile proteins. Given these contradictory findings, we aimed to clarify the role of *Asb2* in muscle mass and strength using a skeletal muscle‐specific *Asb2* knockout (*Asb2* MKO) mouse model. Additionally, we investigate the long‐term effects of *Asb2* on aged muscle, underlying mechanisms on muscle regulation and metabolic effects of *Asb2* MKO mice to better understand its role in muscle function and age‐related metabolic diseases.

**Methods:**

*Asb2* MKO mice were generated using *Acta1*‐Cre recombinase. Body composition was quantified in male and female mice up to 18 months of age. Muscle strength, energy expenditure and glucose metabolism were evaluated using the grip strength test, mitochondrial oxygen consumption measurement, indirect calorimetry and glucose/insulin tolerance tests. Transcriptomic analyses and siRNA studies were performed to elucidate the mechanisms underlying the *Asb2* deletion.

**Results:**

The MKO mice were born healthy and exhibited selective *Asb2* deletion in the skeletal muscle, leaving the cardiac muscle unaffected. This deletion led to an increase in the mass of various skeletal muscles (9%–23%, *p* < 0.05) and improved grip strength (~10%, *p* < 0.05), both of which were sustained throughout the ageing process. The MKO mice also revealed enhanced mitochondrial function, energy expenditure and whole‐body insulin sensitivity. Transcriptomic data supported the muscle phenotype observed in the MKO mice. Notably, desmin, a protein critical for structural integrity and mitochondrial function, was identified as a target protein of the ASB2 E3 ligase.

**Conclusions:**

Skeletal muscle‐specific deletion of *Asb2* led to increased muscle mass and strength, potentially through preservation of desmin levels. These findings suggest that targeting *Asb2* may enhance muscle growth and prevent age‐related muscle decline, with potential benefits for metabolic health, particularly by improving mitochondrial function and insulin sensitivity.

## 1 Introduction

Ageing leads to a gradual decline in muscle mass known as sarcopenia. This condition impairs mobility, increases the risk of falls and fractures and creates a vicious cycle of reduced physical activity and muscle loss [1]. Sarcopenia is a predictor of mortality in older adults [2], and greater muscle mass is linked to reduced all‐cause mortality, emphasizing the importance of maintaining muscle mass for longevity [3]. Additionally, as skeletal muscle comprises approximately 40% of body mass, it plays an important role in whole‐body energy metabolism, regulating glucose, amino acids and lipid homeostasis [4]. Given the increase in metabolic diseases that contribute to morbidity and mortality, preserving skeletal muscle mass is crucial for promoting healthy ageing.

The ankyrin repeat and suppressor of cytokine signalling box protein 2 (*Asb2*) are a member of the *Asb* family, which comprises 18 members characterized by variable numbers of N‐terminal ankyrin repeats. In mammals, the *Asb2* gene encodes two isoforms, *Asb2α* and *Asb2β*. *Asb2α* is expressed in haematopoietic cells and was initially identified as a retinoic acid‐inducible gene involved in inducing differentiation of myeloid leukaemia cells [5]. *Asb2β* is implicated as a muscle‐specific E3 ligase that induces myoblast differentiation in vitro [6]; moreover, it stimulates the degradation of sarcomere‐associated proteins in the cardiac muscle of neonatal mice [7]. In skeletal muscles, follistatin‐induced muscle hypertrophy is associated with reduced expression of *Asb2*, and adeno‐associated virus‐mediated direct local injection of *Asb2* in mouse skeletal muscles reduces muscle fibre size [8], suggesting that *Asb2* is a negative regulator of muscle mass beyond the cardiac muscle. However, a previous study reported that the knockdown of endogenous *Asb2* using shRNAs decreases myoblast fusion and contractile protein expression in C2C12 cells [6]. These mismatched results regarding muscle mass and strength raise questions about the development of treatments that simultaneously increase muscle mass and function by targeting *Asb2*.

Moreover, although previous studies have demonstrated that *Asb2* overexpression can decrease skeletal muscle mass [8], it remains unclear whether *Asb2* deletion promotes muscle growth in vivo, as it has not been successfully investigated. This effect is partly due to the lethal phenotype observed in global *Asb2* knockout mice [9], which exhibit cardiogenesis defects owing to their high expression levels in the cardiac muscle. Consequently, the role of *Asb2* in augmenting skeletal muscle mass and strength remains unclear. Furthermore, its long‐term therapeutic potential in preventing ageing‐associated muscle loss and metabolic disorders remains unknown. In this study, using skeletal muscle‐specific *Asb2* knockout (*Asb2* MKO) mice, we aimed to elucidate the therapeutic role of *Asb2* in enhancing skeletal muscle mass and strength during ageing, as well as the underlying mechanisms involved.

## 2 Methods

### 2.1 Generation of Skeletal Muscle‐Specific *Asb2* Knockout Mice and Diet

The exon 4‐targeted *Asb2* ^2loxP/2loxP^ mice were kindly donated by the Korea Mouse Phenotyping Center (MOP ID: MOP1809004), and the human α‐skeletal actin (*Acta1*)‐Cre mice were purchased from the Jackson Laboratory (RRID: #006149). To generate *Asb2* MKO, *Asb2* ^2loxP/2loxP^ mice were bred with *Acta1*‐Cre mice and backcrossed to a C57BL/6J background over five generations. Animal experiments with *Asb2* MKO mice were conducted using age‐ and sex‐matched littermates *Asb2* ^2loxP/2loxP^ mice as controls (WT). Mice were individually housed in a specific pathogen‐free facility under controlled temperature (22°C ± 2°C), humidity (55% ± 10%) and lighting (12 h light/dark cycle). The mice had free access to water and were fed ad libitum with either a regular chow diet (RC; 5053, Labdiet, St. Louis, MO) or a high‐fat diet (HFD; D12492, Research Diets, New Brunswick, NJ). Animal studies were conducted in the AAALAC‐accredited animal facility at the Lee Gil Ya Cancer and Diabetes Institute of Gachon University. All experimental animal procedures were approved by the Institutional Animal Care and Use Committee of the Lee Gil Ya Cancer and Diabetes Institute of Gachon University (Permission Number: LCDI‐2017‐0118).

### 2.2 Body Composition and Energy Balance Study

The body composition, including fat and lean body mass, of WT and *Asb2* MKO mice was determined using a ^1^H‐nuclear magnetic resonance system (Bruker Optics, Billerica, MA) without anaesthesia. The Comprehensive Lab Animal Monitoring System (CLAMS, Columbus Instruments, Columbus, OH) was utilized to measure various metabolic parameters, including the oxygen consumption rate (V_O2_), carbon dioxide production (V_CO2_), respiratory exchange ratio (RER), energy expenditure, activity and food intake. Following a 24‐h acclimation period, data were wirelessly acquired every 15 min for 48 h.

### 2.3 Grip Strength Test

A digital force gauge (DEFII‐002, Chatillon, Largo, FL) was used for to assess total four‐limb grip strength in mice. In the fed state, the mice were acclimatized to a wire mesh for 5 min, and then, the mouse tail was gently pulled backward in a horizontal manner until the mouse released its grasp on the mesh. The force exerted at the moment of release was recorded as peak tension. Each mouse was tested three times, with a 1‐ to 2‐min break between tests to ensure accurate and consistent measurements.

### 2.4 Intraperitoneal Glucose and Insulin Tolerance Tests

After 4 weeks of HFD feeding, the glucose tolerance test (GTT) and insulin tolerance test (ITT) were conducted. For the GTT, the mice were fasted for 16 h and intraperitoneally injected with a glucose solution (1.5 g/kg body weight). Plasma glucose and insulin concentrations were monitored before injection and at 15, 30, 60, 120 and 180 min after glucose injection. For the ITT, the mice were fasted for 6 h and intraperitoneally injected with insulin (0.75 IU/kg body weight). Plasma glucose concentrations were measured before injection and at 15, 30 and 60 min after insulin injection. Glucose levels were assessed using a glucometer (GM9, Analox Instruments, Stourbridge, United Kingdom), and insulin levels were determined using an insulin ELISA kit (ALPCO, Salem, NH).

### 2.5 C2C12 Cell Culture and Transfection of siRNA

The C2C12 mouse skeletal muscle cell line (CRL‐1772, ATCC, Manassas, VA) was cultured in Dulbecco's modified Eagle's medium (DMEM) supplemented with 10% foetal bovine serum. Myoblast differentiation was initiated upon reaching confluence by replacing the culture medium with DMEM containing 2% horse serum. For *Asb2* knockdown, scrambled control siRNA and si*Asb2* (65256‐3, Bioneer, Daejeon, Korea) were used. C2C12 cells were transfected with 2 μg of siRNA using an electroporator (NEPA21, NePa Gene, Chiba, Japan) and then differentiated into myotubes for 2 days. To examine whether the target protein of ASB2 was degraded through the ubiquitination system, C2C12 myotubes were treated with 10‐μM MG132 for 6 h on Day 2 of differentiation. Myotubes subjected to *Asb2* knockdown or MG132 treatment were harvested for protein extraction and subsequently analyzed by western blotting. All cell culture experiments were independently replicated three times.

### 2.6 RNA Sequencing and Gene Set Enrichment Analysis

RNA sequencing (RNA‐seq) and analysis were conducted by EBIOGEN Inc. Details of RNA sequencing are seen in the Supporting Information. Gene set enrichment analysis (GSEA) was conducted using the open‐source software v4.1.0 (http://software.broadinstitute.org/gsea/index.jsp), and *p* values were calculated by permuting the data 1000 times to identify enriched gene sets. GSEA software produces a nominal *p* value and false discovery rate (*Q* value).

### 2.7 Statistics

Statistical differences between the two groups were assessed using two‐tailed unpaired Student's *t* tests. For comparisons involving more than two groups, a one‐ or two‐way ANOVA (for two independent variables) was conducted, followed by post hoc analysis (Bonferroni, GraphPad Prism 5.0). *p* values < 0.05 were considered significant.

An additional detailed description of haematoxylin–eosin staining, cross‐sectional area analysis, fractionation of muscle tissue, western blotting, quantitative polymerase chain reaction and isolated mitochondrial respiration can be found in the Supporting Information.

## 3 Results

### 3.1 Construction of Skeletal Muscle‐Specific *Asb2* Knockout Mice

The *Asb2* gene is expressed in skeletal muscles and in various tissues, including the heart, in humans [6]. Our data revealed that *Asb2* was abundantly expressed in both skeletal muscle and heart tissues in mice (Figure S1A). To circumvent the lethality observed in whole‐body *Asb2* knockout mice [9], we adopted a strategy using a muscle‐specific Cre recombinase with an *Acta1* (also known as HSA) promoter/enhancer (Figure S1B), which has been reported to selectively target skeletal muscles rather than the heart [10]. The generated *Asb2* MKO mice were born healthy without lethality or abnormalities. The mRNA expression of *Asb2* was reduced only in the skeletal muscle, with no change observed in the heart (Figure S1C). Furthermore, the removal of the *Asb2* gene did not significantly affect the expression of other *Asb* family genes, including *Asb8*, *Asb10*, *Asb11* and *Asb14*, which are highly expressed in both human and mouse skeletal muscles (as indicated by the BioGPS database, http://biogps.org) (Figure S1D).

### 3.2 *Asb2* MKO Mice Exhibited Increased Skeletal Muscle Mass Throughout Their Lifespan, Regardless of Sex

**FIGURE 1:**
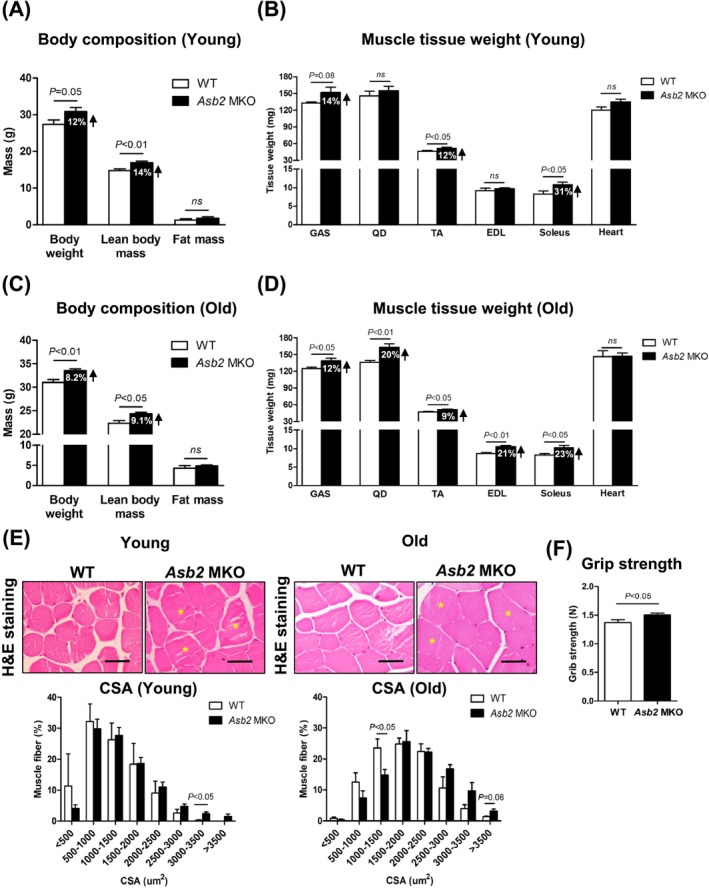
Skeletal muscle‐specific *Asb2* knockout mice revealed increased lean body mass and skeletal muscle tissue weight. Both WT and *Asb2* MKO male mice fasted overnight prior to sacrifice. (A) Body composition of both young 6‐month‐old WT and *Asb2* MKO male mice was measured before fasting (*n* = 6–7 each). (B) Skeletal muscle and heart tissue weights of young 6‐month‐old WT and *Asb2* MKO male mice (*n* = 6–7 each). (C) Body composition of aged 18‐month‐old WT and *Asb2* MKO male mice was measured before fasting (*n* = 6 each). (D) Skeletal muscle and heart tissue weights of aged 18‐month‐old WT and *Asb2* MKO male mice (*n* = 5–6 each). (E) Representative haematoxylin and eosin (H&E)‐stained images and cross‐sectional areas (CSA) of tibialis anterior (TA) muscle from young 6‐month‐old (left) and old 18‐month‐old (right) of WT and *Asb2* MKO mice (*n* = 3–5 each). Asterisks (*) indicate muscle fibres with a CSA greater than 3000 μm^2^. Scale bars represent 40 μm. (F) Grip strength of aged 18‐month‐old WT and *Asb2* MKO male mice. Data are presented as means ± standard error of the mean (SEM). Statistical significance was determined by Student's *t* test. CSA, cross‐sectional area; EDL, extensor digitorum longus; GAS, gastrocnemius; H&E, haematoxylin and eosin; ns, not significant; QD, quadriceps; TA, tibialis anterior.

At 6 months of age (young), *Asb2* MKO male mice exhibited approximately 12% higher body weights than WT (Figure 1A). This increase was primarily attributed to an approximately 14% increase in lean body mass, with no difference in fat mass between the genotypes (Figure 1A). Most muscle tissues, including the gastrocnemius (GAS), tibialis anterior (TA) and soleus muscles, exhibited a 12%–31% increase in *Asb2* MKO male mice compared to WT, whereas the heart weight remained the same (Figure 1B). Notably, the increased muscle mass observed in 6‐month‐old male *Asb2* MKO male mice was sustained throughout the ageing process up to 18 months of age (old) (Figure 1C,D). Similar to the data from 6‐month‐old mice, the increased weight in old *Asb2* MKO male mice was mostly attributed to an increase in lean body mass, regardless of muscle type, with no significant changes observed in the cardiac muscles (Figure 1C,D). These changes were also observed in 18‐month‐old MKO female mice, which exhibited a 17% increase in lean body mass and 8%–19% increases in most skeletal muscle tissues, excluding the heart (Figure S2A,B). Histological analysis of skeletal muscles demonstrated that *Asb2* MKO male mice exhibited an increased proportion of larger fibres (exceeding 3000‐μm^2^ CSA) compared to WT (Figure 1E, left). Notably, muscle fibres with a CSA exceeding 3500 μm^2^ were only observed in the MKO muscle, suggesting that hypertrophic rather than hyperplastic increases occurred in the MKO myofibers (Figure 1E, left). This pattern was also observed at 18 months of age, indicating that the increase in larger fibres persists with ageing (Figure 1E, right). Next, we examined whether the increased muscle mass enhanced muscle strength. At 18 months of age, both male and female *Asb2* MKO mice demonstrated heightened grip strength compared to their WT counterparts (Figures 1F and S2C). Overall, these results suggest that muscle‐specific deletion of *Asb2* leads to an increase in muscle size at a young age and maintains increased muscle mass and strength at an old age in both sexes, indicating a potent preventive role against sarcopenia in vivo.

### 3.3 *Asb2* MKO Mice Exhibited Increased Whole‐Body Energy Expenditure

**FIGURE 2:**
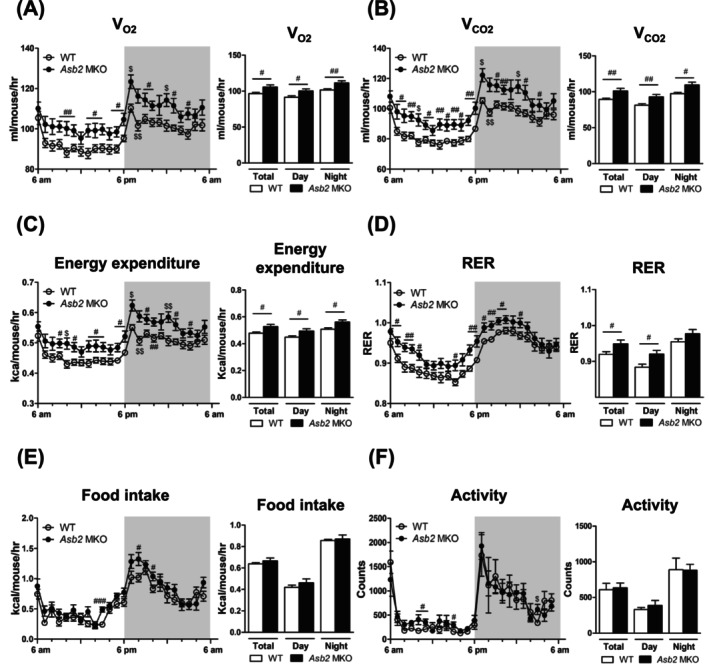
Skeletal muscle‐specific *Asb2* knockout mice revealed increased energy expenditure. Whole‐body energy balance in young 6‐month‐old WT and *Asb2* MKO male mice fed a regular chow diet. (A) Oxygen consumption (V_O2_), (B) carbon dioxide production (V_CO2_), (C) energy expenditure, (D) respiratory exchange ratio (RER), (E) food intake and (F) activity were measured in mice housed in individual metabolic cages for 48 h (*n* = 10 each). Data are presented as means ± standard error of the mean (SEM). ^$^ *p* < 0.05 and ^$$^ *p* < 0.01 by two‐way ANOVA. ^#^ *p* < 0.05, ^##^ *p* < 0.01 and ^###^ *p* < 0.001 by Student's *t* test. RER, respiratory exchange ratio; V_CO2_, carbon dioxide production; V_O2_, oxygen consumption.

Next, to determine whether increased muscle mass owing to *Asb2* deletion contributes to enhanced whole‐body energy expenditure, we conducted indirect calorimetry on 6‐month‐old male mice. As shown in Figure 2, *Asb2* MKO mice demonstrated an overall increase in O_2_ consumption (Figure 2A) and CO_2_ production (Figure 2B) over a 24 h period (time point curve graph in the left panel and daily average graph in the right panel) compared to WT mice. Consequently, energy expenditure was significantly higher in *Asb2* MKO mice than in WT mice (Figure 2C). This phenomenon corresponded with an increase in the respiratory exchange ratio (RER) throughout the 24‐h period, indicating a preference for carbohydrates as the energy source, even during the inactive period, which typically favours lipids as the energy source (Figure 2D). Food intake in *Asb2* MKO mice was not significantly increased; however, a trend towards an increase was observed compared to that in WT mice (Figure 2E). Additionally, *Asb2* MKO mice did not exhibit significant differences in locomotor activity compared with WT mice (Figure 2F). Collectively, *Asb2* MKO mice exhibited an overall increase in V_O2_, V_CO2_, RER and energy expenditure, with these increases consistently observed throughout the day and night under carbohydrate‐enriched conditions.

### 3.4 *Asb2* MKO Mice Displayed Modest Metabolic Advantages When Subjected to an HFD

**FIGURE 3:**
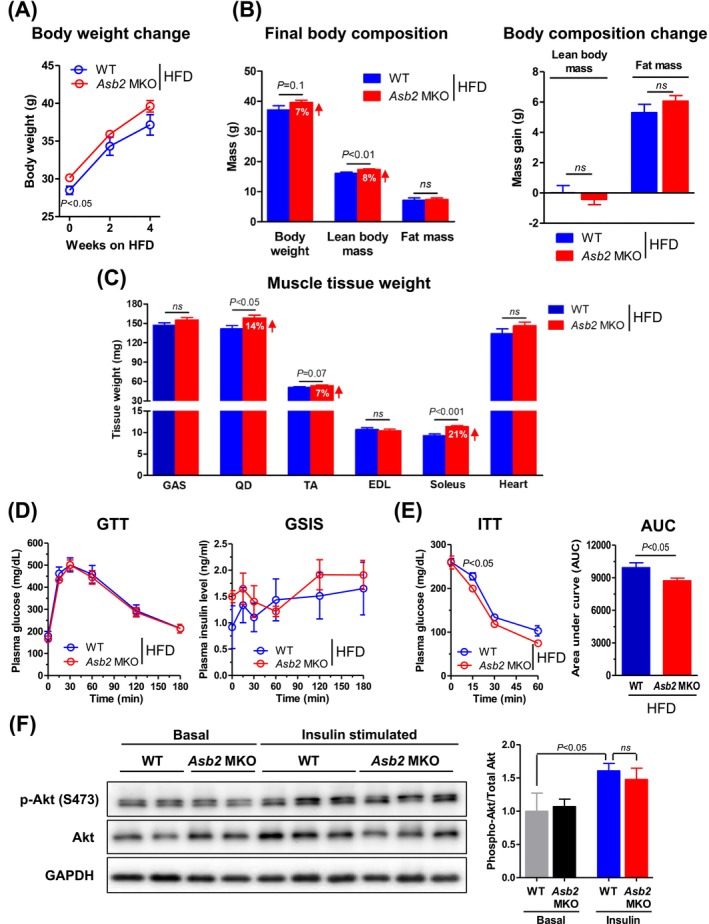
Skeletal muscle‐specific *Asb2* knockout mice displayed modest metabolic advantages when subjected to a high‐fat diet. Six‐month‐old WT and *Asb2* MKO male mice fed a high‐fat diet (HFD) for a period of 4 weeks. (A) Body weight change curve for 4 weeks of HFD feeding (*n* = 10–14 each). (B) Final body weight and body composition measured before fasting (left) and changes in lean body and fat mass before and after 4 weeks of HFD feeding (right) (*n* = 10–14 each). (C, D) Analyses were performed on HFD‐fed WT and *Asb2* MKO male mice that were fasted overnight prior to sacrifice. (C) Skeletal muscle and heart tissue weights (*n* = 10–14 each). (D) Glucose tolerance test (GTT) (left) and glucose‐stimulated insulin secretion (GSIS) (right) were performed on HFD‐fed WT and *Asb2* MKO male mice after overnight fasting (*n* = 4–5 each). (E, F) Insulin tolerance test (ITT) and insulin signalling analysis were performed on HFD‐fed WT and *Asb2* MKO male mice after 6 h of fasting. (E) ITT (left) and area under the curve (AUC) (for 60 min) (right) (*n* = 3–6 each). (F) Representative immunoblots of phospho‐Akt (Ser473), Akt and GAPDH in gastrocnemius (GAS) muscle of WT and *Asb2* MKO mice at 60 min after insulin injection following 4 weeks on HFD (*n* = 3–5 each). Data are presented as means ± standard error of the mean (SEM). Statistical significance was determined by Student's *t* test. AUC, area under curve; EDL, extensor digitorum longus; GAS, gastrocnemius; GAPDH, glyceraldehyde‐3‐phosphate dehydrogenase; GSIS, glucose‐stimulated insulin secretion; GTT, glucose tolerance test; HFD, high‐fat diet; ITT, insulin tolerance test; ns, not significant; QD, quadriceps; TA, tibialis anterior.

To evaluate the influence of increased muscle mass in *Asb2* MKO male mice on potential benefits for metabolic diseases under lipid‐enriched conditions, we subjected these mice to a HFD for 4 weeks. Although the body weight of *Asb2* MKO mice was higher than that of WT mice before HFD feeding, no difference in body weight gain was observed between the two groups during HFD feeding (Figure 3A). After HFD feeding, the increased lean body mass of *Asb2* MKO mice was sustained at approximately 8% higher than that of WT mice (Figure 3B) but slightly decreased compared to the 14% increase observed under RC conditions (Figure 1A). There was no difference in body fat mass gain between the genotypes (Figure 3B). Although increased muscle mass in quadriceps (QD) and soleus muscles of *Asb2* MKO mice was evident after HFD feeding (Figure 3C), that in GAS and TA was less pronounced than that on the RC. Furthermore, *Asb2* MKO mice exhibited no increase in V_O2_, V_CO2_, energy expenditure, RER, food intake or locomotor activity under HFD feeding (Figure S3). These data indicate that although *Asb2* MKO mice showed increased muscle mass on the HFD, it was less than that observed on RC, with minimal impact on whole‐body energy metabolism. Together with data from RC, these results suggest that the metabolic phenotype associated with increased muscle mass in *Asb2* MKO mice is more prominently manifested under carbohydrate‐rich dietary conditions rather than lipid‐enriched environments.

To investigate the role of *Asb2* in glucose metabolism, we conducted GTT and ITT in HFD‐fed male mice. During the GTT, plasma glucose (Figure 3D, left) and insulin concentrations, indicating glucose‐stimulated insulin secretion (Figure 3D, right), were identical between the genotypes. However, during the ITT, there were slight but significant reductions in plasma glucose concentration 15 min after insulin injection (Figure 3E, left); moreover, the area under the curve was significantly decreased in *Asb2* MKO mice (Figure 3E, right), indicating an improvement in whole‐body insulin sensitivity due to muscle‐specific *Asb2* deletion. The improved whole‐body insulin sensitivity observed in *Asb2* MKO mice appeared to be due to an increase in absolute muscle mass rather than an improvement in insulin signalling at the muscle cell level. This conclusion is supported by the comparable induction of Akt phosphorylation at Ser473 in response to insulin in both WT and *Asb2* MKO muscles (Figure 3F). Overall, these results suggest a mild improvement in whole‐body insulin sensitivity in *Asb2* MKO mice, although the augmented muscle mass was insufficient to counteract HFD‐induced obesity or glucose intolerance.

### 3.5 RNA‐Seq Analysis Revealed Enriched Gene Sets Related to Muscle Mass in *Asb2* MKO Mice

**FIGURE 4:**
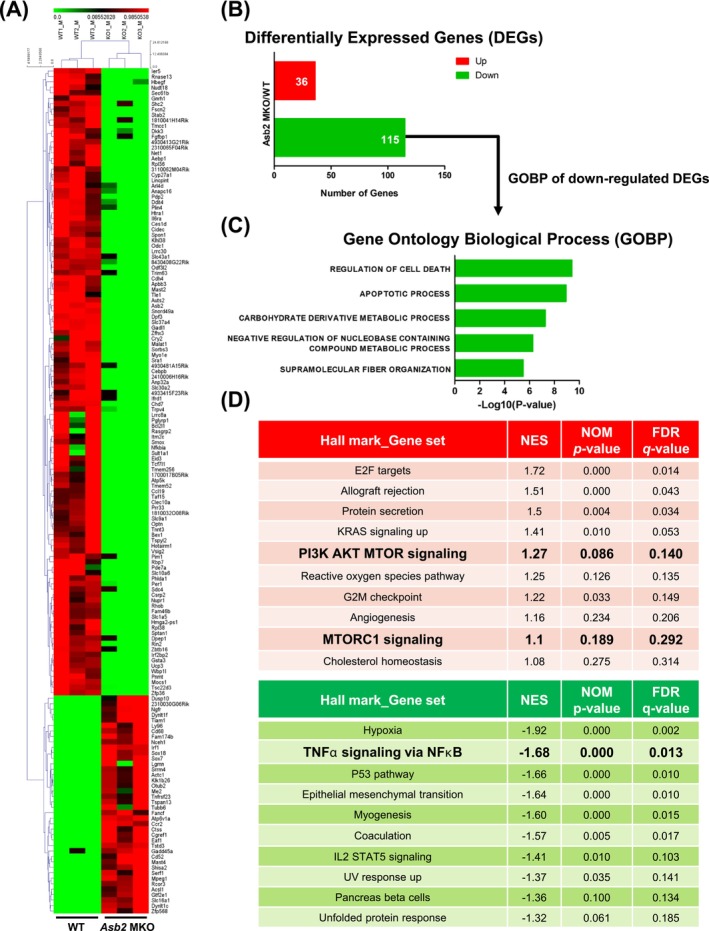
RNA‐Seq analysis revealed enriched gene sets related to muscle mass in skeletal muscle‐specific *Asb2* knockout mice. RNA‐Seq analyses using gastrocnemius (GAS) muscles of young 6‐month‐old WT and *Asb2* MKO male mice fasted overnight prior to sacrifice (*n* = 3 each). (A) Hierarchically clustered differentially expressed genes (DEGs) presented as heat maps according to the *Z* score (red indicates upregulated genes; green indicates downregulated genes). (B) Number of DEGs. (C) Gene Ontology Biological Processes (GOBP) analyses of the transcripts derived from RNA‐seq revealed statistically significant differences using the Molecular Signatures Database (MSigDB). (D) Gene set enrichment analysis (GSEA) on the hallmark gene sets for RNA‐seq analyses between *Asb2* MKO versus WT (red indicates gene sets for upregulated genes; green indicates gene sets for downregulated genes). DEGs, Differentially expressed genes; GAS, gastrocnemius; GOBP, gene ontology biological processes; GSEA, gene set enrichment analysis; MSigDB, molecular signatures database.

Next, we conducted RNA‐seq analysis of WT and *Asb2* MKO muscles from male mice to understand the transcriptional changes involved in altering muscle mass linked to the *Asb2* gene under RC conditions. We identified 151 differentially expressed genes (DEGs), with 36 upregulated and 115 downregulated genes in the *Asb2* MKO muscle compared to those in the WT muscle (Figure 4A,B and Table S2). Although Gene Ontology analysis did not identify specific biological process terms associated with the 36 upregulated genes, five biological process terms were identified with the 115 downregulated genes, including ‘regulation of cell death’ and ‘apoptosis process’ as the top two associated biological processes (Figure 4C). Cell death and apoptosis are increasing features of sarcopenia and muscle atrophy [11]; thus, downregulation of these processes could explain the phenotypes observed in *Asb2* MKO muscle. Consistently, GSEA revealed the upregulation of enriched pathways involved in protein synthesis, such as ‘phosphatidylinositol 3‐kinase‐Akt‐mTOR (PI3K‐Akt‐mTOR) signalling’ and ‘mTORC1 signalling’ in *Asb2* MKO muscle (Figure 4D). Activation of the PI3K–Akt–mTOR and mTORC1 pathways occurs in response to loading and growth factors, such as insulin and insulin‐like growth factor, which prevent the loss of skeletal muscle mass [4]. GSEA also identified downregulated pathways in *Asb2* MKO mice, including the ‘tumour necrosis factor‐α (TNF‐α) signalling via nuclear factor‐κB (NF‐κB)’ gene set (Figure 4D). TNF‐α has been implicated as a possible mediator of muscle catabolism via stimulation of the ubiquitin‐proteasome system and plays a role in the induction of cachexia in animal studies [12]. NF‐κB, which regulates the transcription of genes involved in cell death and immune responses, has been reported to mediate the protein catabolism induced by TNF‐α in differentiated skeletal muscle myotubes [13]. Western blot analyses revealed significantly increased phosphorylation of Akt (Ser473 and Thr308), along with a trend towards increased phosphorylation of p70 ribosomal S6 kinase (p70S6K) (Thr389), which are key components of the protein synthesis pathway (Figure S4A–E). In contrast, the phosphorylation of NF‐κB p65 (Ser536) involved in the protein degradation process showed a decreasing trend in *Asb2* MKO mice (Figure S4A,F). Overall, these findings suggest that protein synthesis processes are increased and protein degradation processes are reduced in skeletal muscle owing to *Asb2* deletion, supporting the augmented muscle mass phenotype of *Asb2* MKO mice.

### 3.6 *Asb2* MKO Mice Revealed Increased Expression of Desmin in Skeletal Muscles

**FIGURE 5:**
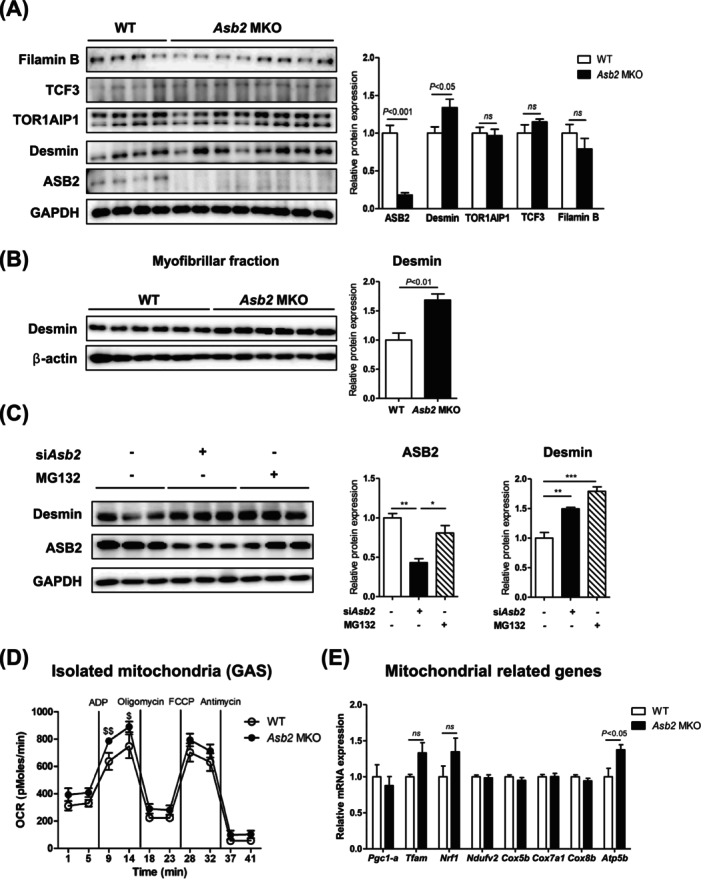
Skeletal muscle‐specific *Asb2* knockout mice revealed increased protein expression of desmin in skeletal muscle. (A) Representative immunoblots of ankyrin repeat and suppressor of cytokine signalling box protein 2 (ASB2) and its target proteins' expression in the tibialis anterior (TA) muscle of 6‐month‐old WT and *Asb2* MKO male mice fasted overnight prior to sacrifice (left). Bar charts display the quantification of optical densities of blot bands for filamin B, transcription factor 3 (TCF3), torsin 1A interacting protein 1 (TOR1AIP1), desmin and ASB2 (right) (*n* = 8 each). (B) Immunoblots of desmin protein expression in myofibrillar fraction isolated from gastrocnemius (GAS) muscle of young 6‐month‐old WT and *Asb2* MKO mice fasted overnight prior to sacrifice (left). Bar charts show the quantification of optical densities of blot bands for desmin (right) (*n* = 6 each). (C) Representative immunoblots of ASB2 and desmin protein expression in *Asb2* knockdown or MG132 treated myotube cells (left). Bar charts show the quantification of optical densities of blot bands for ASB2 and desmin (right) (*n* = 3 each). (D) Respiration of mitochondria isolated from GAS muscle of young 6‐month‐old WT and *Asb2* MKO mice fasted overnight prior to sacrifice (*n* = 4 each). (E) mRNA expression of mitochondrial‐related genes in GAS muscle of young 6‐month‐old WT and *Asb2* MKO mice fasted overnight prior to sacrifice (*n* = 4–8 each). Data are represented as means ± standard error of the mean (SEM). ^$^ *p* < 0.05 and ^$$^ *p* < 0.01 by two‐way ANOVA (D). *p < 0.05, **p < 0.01 and ***p < 0.001 by one‐way ANOVA (C). Statistical significance was determined by Student's *t* test (A, B, E). ADP, adenosine diphosphate; ASB2, ankyrin repeat and suppressor of cytokine signalling box protein 2; *Atp5b*, ATP synthase F1 subunit beta; Cox, cytochrome C oxidase subunit; FCCP, carbonyl cyanide‐4‐(trifluoromethoxy) phenylhydrazone; GAPDH, glyceraldehyde‐3‐phosphate dehydrogenase; GAS, gastrocnemius; *Ndufv2*, NADH–ubiquinone oxidoreductase core subunit V2; *Nrf1*, nuclear respiratory factor 1; ns, not significant; OCR, oxygen consumption rate; *Pgc1‐α*, peroxisome proliferator‐activated receptor 1 alpha; TA, tibialis anterior; TCF3, transcription factor 3; *Tfam*, mitochondrial transcription factor A; TOR1AIP1, torsin 1A interacting protein 1.

To investigate whether ASB2 target proteins contribute to the increased muscle mass observed in *Asb2* MKO mice, we examined their expression in TA muscle. Specifically, we assessed proteins previously identified as ASB2 substrates in various in vitro studies including filamin B [6], transcription factor 3 (TCF3) [14], desmin [7] and torsin 1A interacting protein 1 (TOR1AIP1) [15]. Among these proteins, only desmin expression correlated with *Asb2* deletion in the MKO muscle, whereas TOR1AIP1, TCF3 and filamin B remained unchanged (Figure 5A). As desmin is primarily localized around the Z‐disc of myofibrils [16], we also found desmin expression was increased in the myofibril fraction of *Asb2* MKO compared to WT (Figure 5B). Furthermore, desmin protein expression was markedly increased upon *Asb2* knockdown, reaching levels comparable to those seen in proteasome inhibitor MG132‐treated cells (Figure 5C). These findings suggest that desmin is degraded under normal conditions predominantly via ubiquitin‐mediated proteasomal degradation and that ASB2 likely contributes to this regulation as an E3 ubiquitin ligase.

Given desmin's crucial role in structural integrity [17], preventing muscle wasting [18] and its association with mitochondrial dysfunction [19] and impaired ATP‐dependent contraction in myopathies [20], we assessed mitochondrial function in *Asb2* MKO muscle. Mitochondria isolated from *Asb2* MKO muscle showed increased oxygen consumption rate, particularly in the section that responded to ADP‐stimulated ATP production (Figure 5D), along with increased expression of mitochondrial ATP synthase F1 subunit beta (*Atp5b*) gene (Figure 5E). Overall, these data suggest that *Asb2* deletion may increase muscle mass and enhance muscle strength and quality by stabilizing desmin and improving mitochondrial function.

## 4 Discussion

In the present study, we provide new insights into the role of *Asb2* in regulating skeletal muscle mass and its effects on energy metabolism throughout the lifespan using a skeletal muscle‐specific *Asb2* KO model, excluding the cardiac muscle. We demonstrated, for the first time, that targeted deletion of the *Asb2* gene increases skeletal muscle mass in vivo, leading to increases in energy expenditure and grip strength, regardless of sex. These effects were sustained throughout ageing, highlighting *Asb2* as a key negative regulator of muscle mass. These findings are particularly relevant for ageing‐ and metabolic‐related diseases such as sarcopenia and cancer cachexia, which involve loss of muscle mass and strength [21]. Because muscle loss reduces physical activity and worsen muscle wasting [22], whereas greater muscle strength improves survival in older cancer patients undergoing chemotherapy [23], these findings suggest that *Asb2* could be a potential therapeutic target for enhancing and maintaining both muscle mass and strength.

This study also revealed the potential underlying mechanisms contributing to the augmentation of both muscle mass and strength. We observed that desmin exhibited a pattern of expression opposite to that of ASB2 in skeletal muscle, with desmin protein levels significantly increased in *Asb2* MKO mice. Similarly, both siRNA‐mediated knockdown of *Asb2* or treatment with the proteasome inhibitor MG132 in myocytes also elevated desmin protein levels (Figure 5C). The consistent elevation of desmin following both *Asb2* knockdown and proteasome inhibition highlights the importance of ubiquitination pathways in desmin homeostasis. Although further studies are needed to determine whether ASB2 exclusively targets desmin as a mechanism to regulate skeletal muscle mass, our findings suggest that modulating the activity of E3 ligase, such as ASB2, could be an effective strategy to maintain appropriate desmin protein levels in skeletal muscle, which are essential for preserving structural and cellular integrity muscle [17, 24].

These results are consistent with a previous report indicating that ASB2 colocalizes with desmin and triggers its proteasomal degradation in neonatal mouse cardiac myocytes [7]. Given desmin's central role in preserving sarcomere architecture, cytoskeletal integrity and mechanical stability, the preservation of desmin may contribute directly to the enhanced muscle strength observed in *Asb2* MKO mice. Indeed, desmin knockout mice are weaker and fatigue more easily and are more susceptible to contraction‐mediated skeletal muscle damage [25]. Desmin degradation during systemic catabolic states promotes the destruction of contractile myofibrils and contributes to skeletal muscle atrophy [26]. Additionally, desmin plays a protective role against muscle wasting in dystrophin knockout mice [18] and helps counteract TNF‐α‐induced apoptosis in muscle [27], consistent with our RNAseq and protein expression results (Figures 4 and S4). Desmin is also crucial for linking contractile myofibrils to the sarcolemma and mitochondria [16], stabilizing and positioning mitochondria to support their function [19]. Desmin mutations can cause mitochondrial dysfunction [19], and desmin helps utilize mitochondrial ATP for muscle contraction [20]. In this study, mitochondrial oxygen consumption for ATP generation increased in *Asb2* MKO mice (Figure 5D), likely due to enhanced desmin–mitochondria interaction. This suggests that the involvement of desmin in mitochondrial function contributes to the enhanced grip strength and energy expenditure observed in *Asb2* MKO mice.

In addition to desmin, transcriptomic analysis identified *Ddit4* as the most downregulated gene in *Asb2* MKO muscle. *Ddit4* is known to the promote muscle atrophy by inhibiting mTORC1 activity [28] and implicated in muscle contraction [29]. Downregulation of *Ddit4* activates mTORC1 and protects against muscle loss in atrophy and cachexia conditions [30, 31, 32]. Consistent with these, we observed enhanced mTOR signalling in *Asb2* MKO mice both at the transcriptomic and protein level, likely due to reduced *Ddit4* expression. Although the interplay between *Asb2* and *Ddit4* requires further investigation, reduced *Ddit4* may synergize with desmin stabilization to increase muscle mass in *Asb2* MKO mice. Additionally, transcriptomic profiling revealed downregulation of genes involved in ‘carbohydrate derivative metabolic processes’ and an upregulation of those related to ‘cholesterol homeostasis’, suggesting metabolic adaptations secondary to increased muscle mass. Especially, upregulation of cholesterol homeostasis genes may help maintain membrane integrity or signalling under increased anabolic activity [33], highlighting broader metabolic effects of *Asb2* deletion that warrant further investigation.

Although this study uncovered new in vivo roles and underlying mechanisms of *Asb2* deletion, its metabolic effects were less pronounced than expected. Despite the observed enhancement of whole‐body energy metabolism in *Asb2* MKO mice (Figure 2), no significant reduction in body fat mass was noted in either RC‐fed or aged mice (Figures 1 and S2). Moreover, the increased energy expenditure disappeared after HFD feeding, narrowing the differences in lean body mass between WT and *Asb2* MKO mice (Figures 3 and S3). One possible explanation for this discrepancy is that the extent of muscle mass gain in *Asb2* MKO mice, ranging from 9% to 23% depending on age and muscle type, may be insufficient to significantly impact adiposity in the absence of exercise or dietary intervention. Previous studies have reported that even approximately a 30% increase in skeletal muscle mass does not necessarily result in fat loss [34, 35] and that marked reductions in body fat mass were observed only in models with extreme muscle hypertrophy, such as myostatin knockout mice (twofold increase) [36] or myostatin‐follistatin combined mice (fourfold increase) [37]. In this context, our data suggest that moderate muscle mass augmentation, as observed in *Asb2* MKO mice, may not alone be sufficient to reduce body fat mass. Alternatively, although the average food intake did not differ between WT and *Asb2* MKO mice, indirect calorimetry revealed time‐specific elevations in food consumption in *Asb2* MKO mice. This subtle increase in energy intake may have further offset the fat‐reducing capacity of elevated energy expenditure.

The clinical relevance of *Asb2* has been evident in the Gene Expression Omnibus (GEO) database, but contrary to our expectation, *Asb2* expression was decreased in the vastus lateralis skeletal muscles of patients with myotonic dystrophy type 2 (GEO accession GDS5276). Our investigation in the skeletal muscle of mitochondrial polymerase γ mutant mice (*Polg* mut), an ageing‐associated myopathy model [38], revealed a significant decrease in lean body mass (Figure S5A) and *Asb2* mRNA expression in QD muscle (Figure S5B) compared to age‐matched WT mice. There was a positive correlation between *Asb2* expression and lean body mass in individual *Polg* mut mice (Figure S5C), similar to that observed in human myotonic dystrophy. Unlike MKO mice in which the *Asb2* gene is deleted from birth, the reduced expression of the *Asb2* gene observed in muscles affected by primary conditions such as myotonic dystrophy or mitochondrial myopathy may represent a compensatory mechanism aimed at protecting against muscle loss. This underscores the importance of considering the sequential order of compensatory actions in vivo rather than relying solely on a snapshot of gene expression.

Although our study provides new insights into the physiological and mechanistic roles of *Asb2* in skeletal muscle, several limitations remain. First, we could not assess the role of *Asb2* deletion at 24 months of age, when sarcopenia becomes more prominent. Second, because the *Asb2* gene was deleted from early development using a skeletal muscle‐specific Cre model, it remains unclear whether similar effects would be observed if *Asb2* expression were suppressed in later after development. Additional studies using inducible knockout models would be necessary to address this. Third, it is not clear whether the improved muscle strength in *Asb2* MKO mice are results from increased muscle size or enhanced intrinsic contractile function. Direct assessments of muscle fibre contractility would be required to clarity this.

In conclusion, this study demonstrated that deletion of the *Asb2* gene in skeletal muscle increased muscle mass and improved muscle strength, both of which were sustained up to 18 months of age (old) in mice, regardless of sex. These effects were accompanied by enhanced whole‐body energy expenditure and improved insulin tolerance. Mechanistically, *Asb2* appears to modulate muscle mass via regulation of desmin protein stability. Collectively, these findings suggest that targeting *Asb2* in a skeletal muscle‐specific manner may offer a novel therapeutic strategy to combat sarcopenia by promoting both skeletal muscle mass and strength.

## Ethics Statement

The authors certify that they comply with the ethical guidelines for publication in the *Journal of Cachexia, Sarcopenia and Muscle*.

## Conflicts of Interest

The authors declare no conflicts of interest.

## Supporting information


**Figure S1** Construction of skeletal muscle‐specific *Asb2* knockout mice. (A) Tissue expression pattern of the *Asb2* gene in 6‐month‐old C57BL/6 J male mice fasted overnight prior to sacrifice (*n* = 4 each). (B) Generation of skeletal muscle‐specific *Asb2* knockout (*Asb2* MKO) mouse models, *Asb2* ^2loxP/2loxP^ mice (Control; WT) were crossed with the actin alpha 1, skeletal muscle (*Acta1*)‐Cre mice to generate *Asb2* MKO mice. (C) *Asb2* mRNA levels in different tissues obtained from 6‐month‐old WT and *Asb2* MKO male mice fasted overnight prior to sacrifice (*n* = 3~4 each). (D) mRNA levels of *Asb* family genes, which are abundantly expressed in muscle, in the gastrocnemius muscle of 6‐month‐old C57BL/6J male mice fasted overnight prior to sacrifice (*n* = 4 each). Data are represented as means ± standard error of the mean (SEM). Statistical significance was determined using Student’s *t* test. Not significant, ns; Ankyrin repeat and suppressor of cytokine signaling box protein, *Asb*; Gastrocnemius, GAS; White adipose tissue, WAT; Brown adipose tissue, BAT; Actin alpha 1, skeletal muscle, *Acta1*; Tibialis anterior, TA; Quadriceps, QD.
**Figure S2** Deletion of the skeletal muscle‐specific *Asb2* gene increased skeletal muscle tissue weight and grip strength regardless of sex. (A) Body composition of aged 18‐month‐old WT and *Asb2* MKO female mice measured before fasting (*n* = 6~7 each). (B) Skeletal muscle and heart tissue weights of aged 18‐month‐old WT and *Asb2* MKO female mice fasted overnight prior to sacrifice (*n* = 6~7 each). (C) Grip strength of 18‐month‐old WT and *Asb2* MKO female mice fed a regular chow diet (n = 6~7 each). Data are represented as means ± standard error of the mean (SEM). Statistical significance was determined using Student’s *t* test. Not significant, ns; Gastrocnemius, GAS; Quadriceps, QD; Tibialis anterior, TA; Extensor digitorum longus, EDL.
**Figure S3** Energy expenditure of skeletal muscle‐specific *Asb2* knockout mice under high‐fat diet conditions. Whole‐body energy balance in 6‐month‐old WT and *Asb2* MKO male mice fed a high‐fat diet (HFD) for 4 weeks. (A) Oxygen consumption (V_O2_), (B) carbon dioxide production (V_CO2_), (C) energy expenditure, (D) respiratory exchange ratio (RER), (E) food intake, and (F) activity were measured in mice housed in individual metabolic cages for 48 hours (*n* = 10 each). Data are represented as means ± standard error of the mean (SEM) and expressed per mouse.
**Figure S4** Skeletal muscle‐specific *Asb2* knockout mice exhibited a tendency toward increased protein synthesis and decreased protein degradation signaling in gastrocnemius muscle. (A‐F) Representative immunoblots of protein synthesis and protein degradation signaling in the gastrocnemius (GAS) muscle of 6‐month‐old WT and *Asb2* MKO male mice fasted overnight prior to sacrifice. The p‐Akt (Thr308, Ser473), p‐mTOR (Ser2448), p‐p70S6K (Thr389), p‐NF‐κB P65 (Ser536) levels were analyzed by Western blotting, and quantified (*n* = 5 each). Data are represented as means ± standard error of the mean (SEM). Statistical significance was determined by Student’s *t* test. Not significant, ns; Ankyrin repeat and suppressor of cytokine signaling box protein 2, ASB2; Gastrocnemius, GAS; Mammalian target of rapamycin, mTOR; p70 ribosomal S6 kinase, p70S6K; Nuclear factor‐κB, NF‐κB; Glyceraldehyde‐3‐phosphate dehydrogenase, GAPDH.
**Figure S5** *Asb2* expression is associated with myopathy. (A) Body weight and lean body mass of 6‐month‐old WT and mitochondrial polymerase γ mutant mice (*Polg* mut) male mice measured before fasting (*n* = 6 each). (B) Ankyrin repeat and suppressor of cytokine signaling box protein (*Asb2*) mRNA expression in the quadriceps muscle obtained from 6‐month‐old WT and *Polg* mut male mice fasted overnight prior to sacrifice (n = 6 each). (C) Correlation of *Asb2* mRNA levels and lean body mass in 6‐month‐old WT and *Polg* mut male mice (*n* = 18). Data are represented as means ± standard error of the mean (SEM). Statistical significance was determined using Student’s *t* test. Mitochondrial polymerase γ, *Polg*; Body weight, BW; Lean body mass, LBM; Ankyrin repeat and suppressor of cytokine signaling box protein 2, *Asb2*.

**Table S1** Primers and antibodies used in qRT‐PCR and Western blot.

**Table S2** List of differentially expressed genes in RNA‐seq.

**Table S3** List of differentially expressed genes for Biological Processes using Gene Ontology.
