# Supplementary material for: Skeletal Muscle‐Specific Deletion of E3 Ligase Asb2 Enhances Muscle Mass and Strength
Source: J Cachexia Sarcopenia Muscle. 2025 Jul 10;16(4):e70007. doi: 10.1002/jcsm.70007 (PMC12246383; doi:10.1002/jcsm.70007)
Supplement: Supplementary file 1 — Figure S1 Construction of skeletal muscle‐specific Asb2 knockout mice. (A) Tissue expression pattern of the Asb2 gene in 6‐month‐old C57BL/6 J male mice fasted overnight prior to sacrifice (n = 4 each). (B) Generation of skeletal muscle‐specific Asb2 knockout (Asb2 MKO) mouse models, Asb2 2loxP/2loxP mice (Control; WT) were crossed with the actin alpha 1, skeletal muscle (Acta1)‐Cre mice to generate Asb2 MKO mice. (C) Asb2 mRNA levels in different tissues obtained from 6‐month‐old WT and Asb2 MKO male mice fasted overnight prior to sacrifice (n = 3~4 each). (D) mRNA levels of Asb family genes, which are abundantly expressed in muscle, in the gastrocnemius muscle of 6‐month‐old C57BL/6J male mice fasted overnight prior to sacrifice (n = 4 each). Data are represented as means ± standard error of the mean (SEM). Statistical significance was determined using Student’s t test. Not significant, ns; Ankyrin repeat and suppressor of cytokine signaling box protein, Asb; Gastrocnemius, GAS; White adipose tissue, WAT; Brown adipose tissue, BAT; Actin alpha 1, skeletal muscle, Acta1; Tibialis anterior, TA; Quadriceps, QD. Figure S2 Deletion of the skeletal muscle‐specific Asb2 gene increased skeletal muscle tissue weight and grip strength regardless of sex. (A) Body composition of aged 18‐month‐old WT and Asb2 MKO female mice measured before fasting (n = 6~7 each). (B) Skeletal muscle and heart tissue weights of aged 18‐month‐old WT and Asb2 MKO female mice fasted overnight prior to sacrifice (n = 6~7 each). (C) Grip strength of 18‐month‐old WT and Asb2 MKO female mice fed a regular chow diet (n = 6~7 each). Data are represented as means ± standard error of the mean (SEM). Statistical significance was determined using Student’s t test. Not significant, ns; Gastrocnemius, GAS; Quadriceps, QD; Tibialis anterior, TA; Extensor digitorum longus, EDL. Figure S3 Energy expenditure of skeletal muscle‐specific Asb2 knockout mice under high‐fat diet conditions. Whole‐body energy balance [file JCSM-16-e70007-s003.docx]

**Supporting information**

**Title: Skeletal muscle-specific deletion of E3 ligase Asb2 enhances muscle mass and strength**

**Material and methods**

**Hematoxylin-eosin staining and cross-sectional area analysis**

The tibialis anterior (TA) skeletal muscle tissue from overnight-fasted mice was fixed in 10% formalin and embedded in paraffin. Paraffin-embedded tissues were cut into 5 μm sections. TA sections were stained with hematoxylin and eosin (H&E) following a series of procedures, including deparaffinization and rehydration. Five images were randomly captured at 200× **total** magnification using an OLYMPUS CX31 biological microscope and Zen 2.3 software (Carl Zeiss Microscopy, Oberkochen, Germany), and the cross-sectional area (CSA) of **~200** muscle fibers **per mouse** was determined using ImageJ software (National Institutes of Health).

**Fractionation of muscle tissue**

Mouse GAS muscles from overnight-fasted mice were homogenized on ice for 30 s in 19 volumes of homogenization buffer (20 mM Tris-HCl, pH 7.2, 5 mM EGTA, 100 mM KCl, and 1% Triton X-100), and incubated for 1 h with gentle agitation at 4 °C. Myofibrils were isolated by centrifugation at 3,000 × *g* for 30 min at 4 °C. The myofibirllar pellet was washed twice in wash buffer (20 mM Tris⋅HCl pH 7.2, 100 mM KCl, and 1 mM DTT), and after the final centrifugation (3,000 × *g* for 10 min at 4 °C) was resuspended in storage buffer (20 mM Tris⋅HCl, pH 7.2, 100 mM KCl, 1 mM DTT, and 20% glycerol) and kept at −80 °C. All buffers contained a protease inhibitor cocktail (Sigma-Aldrich, St. Louis, MA) and a phosphatase inhibitor cocktail (Sigma-Aldrich, St. Louis, MA). Isolated myofibrils were separated on SDS-PAGE for western blotting.

**Western blotting**

For protein extraction, differentiated C2C12 cells and snap-frozen tissues from overnight-fasted mice were homogenized in liquid nitrogen. RIPA buffer supplemented with a protease inhibitor cocktail (Sigma-Aldrich, St. Louis, MA) and a phosphatase inhibitor cocktail (Sigma-Aldrich, St. Louis, MA) were added to the homogenized tissue, followed by further homogenization and a 30 min incubation on ice. After centrifugation at 16,000 × *g* for 20 min, the supernatant was used for immunoblotting. The information on antibodies are shown in Table S1.

**Quantitative polymerase chain reaction**

To extract RNA, TRIzol reagent (Life Technologies, Carlsbad, CA) was used to homogenize snap-frozen tissues from overnight-fasted mice. Subsequent steps involved chloroform separation, isopropanol precipitation, and centrifugation. The isolated RNA was reverse-transcribed using a TOPscript™ RT kit (Enzynomics, Daejeon, Korea). Quantitative real-time PCR analysis, conducted with ABI7300 and TOPreal™ quantitative polymerase chain reaction 2X PreMIX (Enzynomics, Daejeon, Korea), was used to normalize the relative mRNA expression of each target to that of *Gapdh*. Table S1 provides the primers used in this study.

**RNA sequencing**

RNA sequencing (RNA-seq) and analysis were conducted by EBIOGEN Inc. mRNA isolation was performed using a Poly(A) RNA Selection Kit (LEXOGEN, Greenland, NH) and cDNA synthesis and shearing were performed on the isolated mRNAs. Illumina indexes 1~12 were employed for indexing, followed by PCR enrichment. Libraries were created from total RNA using the NEBNext Ultra II Directional RNA-Seq Kit (New England BioLabs, Ipswich, MA), and fragment sizes were assessed using the TapeStation HS D1000 Screen Tape (Agilent Technologies, Santa Clara, CA). Quantification was performed using the StepOne Real-Time PCR System (Life Technologies, Carlsbad, CA). High-throughput sequencing was performed as paired-end 100 sequencing using a NovaSeq 6000 (Illumina, San Diego, CA). FastQC was employed for raw data quality control; adapters and low-quality reads (< Q20) were removed using FASTXTrimmer and BBMap software. Trimmed reads were mapped to the reference genome using TopHat software. Gene expression levels, measured as fragments per kilobase per million reads (FPKM), were estimated using Cufflinks. Quantile normalization was applied to the FPKM values, and data mining was performed using ExDEGA (Ebiogen, Seoul, Korea).

**Isolated mitochondrial respiration**

Skeletal muscle mitochondria were isolated from 6-month-old WT and *Asb2* MKO mice, and the oxygen consumption rate was measured using an XF24 analyzer (Agilent, Santa Clara, CA). The gastrocnemius (GAS) muscle was rapidly removed from overnight-fasted mice and homogenized with a dounce homogenizer in isolation buffer (215 mM mannitol, 75 mM sucrose, 0.1% BSA, 1 mM EGTA, 20 mM HEPES, pH 7.2). Then, the homogenate was centrifuged at 800 × g for 10 min. The supernatant was centrifuged again at 10,000 × *g* for 10 min, and the mitochondrial pellet was used for downstream analysis. Mitochondria (10 μg/mL) isolated from the GAS muscle were added to each well with respiration assay buffer (220 mM mannitol, 70 mM sucrose, 10 mM KH2PO4, 5 mM MgCl2, 1 mM EGTA, 25 mM MOPS, 0.2% BSA, pH 7.2) supplemented with substrates (10 mM pyruvate, 2.5 mM, malate, 2.5 mM glutamate, and 5 mM succinate). Reagents (Final working concentrations: 1 mM ADP, 1 μg/mL oligomycin, 1 mM carbonyl cyanide p-trifluoromethoxyphenylhydrazone, and 2 mM antimycin A) were diluted in respiration assay buffer and sequentially loaded into the ports of the flux plate.

**Supplementary Figure**

**
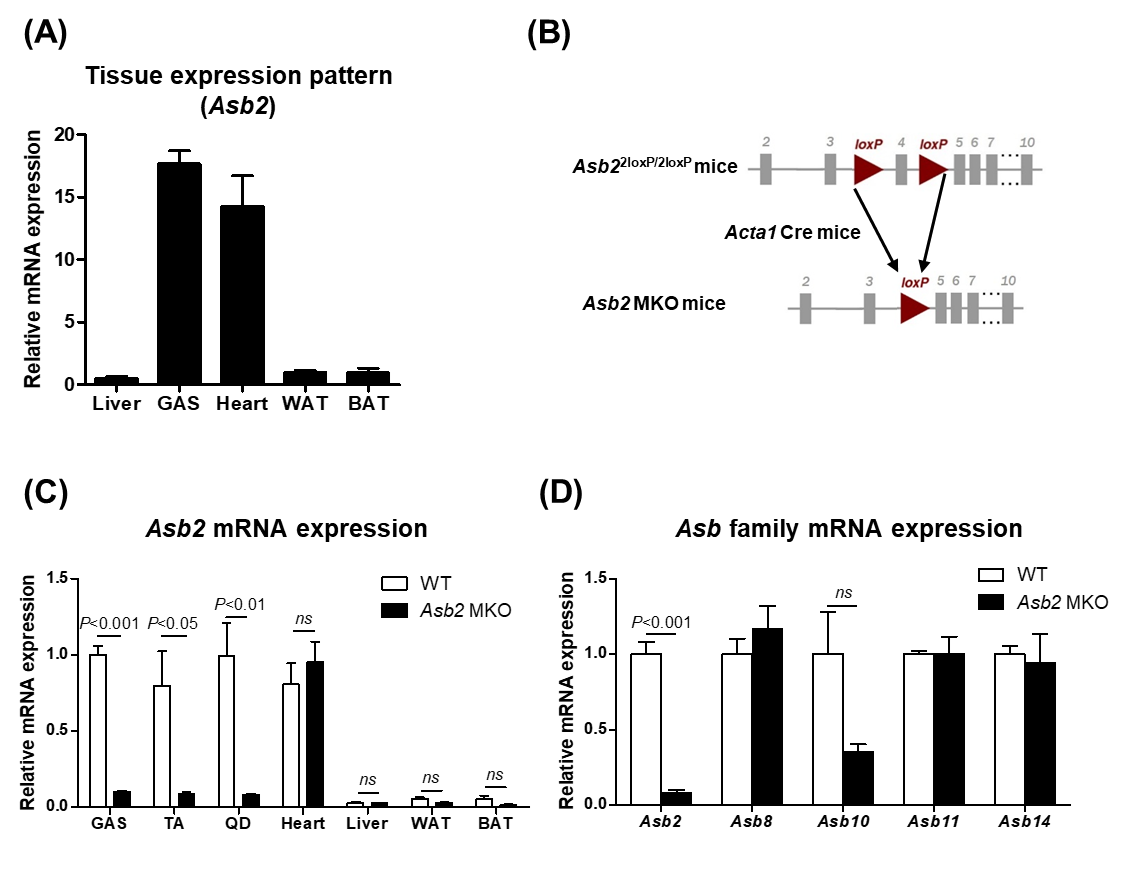
**

**Supplementary Figure S1. Construction of skeletal muscle-specific *Asb2* knockout mice**

(A) Tissue expression pattern of the *Asb2* gene in 6-month-old C57BL/6J male mice fasted overnight prior to sacrifice (n = 4 each). (B) Generation of skeletal muscle-specific *Asb2* knockout (*Asb2* MKO) mouse models, *Asb2*^2loxP/2loxP^ mice (Control; WT) were crossed with the actin alpha 1, skeletal muscle (*Acta1*)-Cre mice to generate *Asb2* MKO mice. (C) *Asb2* mRNA levels in different tissues obtained from 6-month-old WT and *Asb2* MKO male mice fasted overnight prior to sacrifice (n = 3~4 each). (D) mRNA levels of *Asb* family genes, which are abundantly expressed in muscle, in the gastrocnemius muscle of 6-month-old C57BL/6J male mice fasted overnight prior to sacrifice (n = 4 each). Data are represented as means ± standard error of the mean (SEM). Statistical significance was determined using Student’s *t* test. Not significant, ns; Ankyrin repeat and suppressor of cytokine signaling box protein, *Asb*; Gastrocnemius, GAS; White adipose tissue, WAT; Brown adipose tissue, BAT; Actin alpha 1, skeletal muscle, *Acta1*; Tibialis anterior, TA; Quadriceps, QD.

**
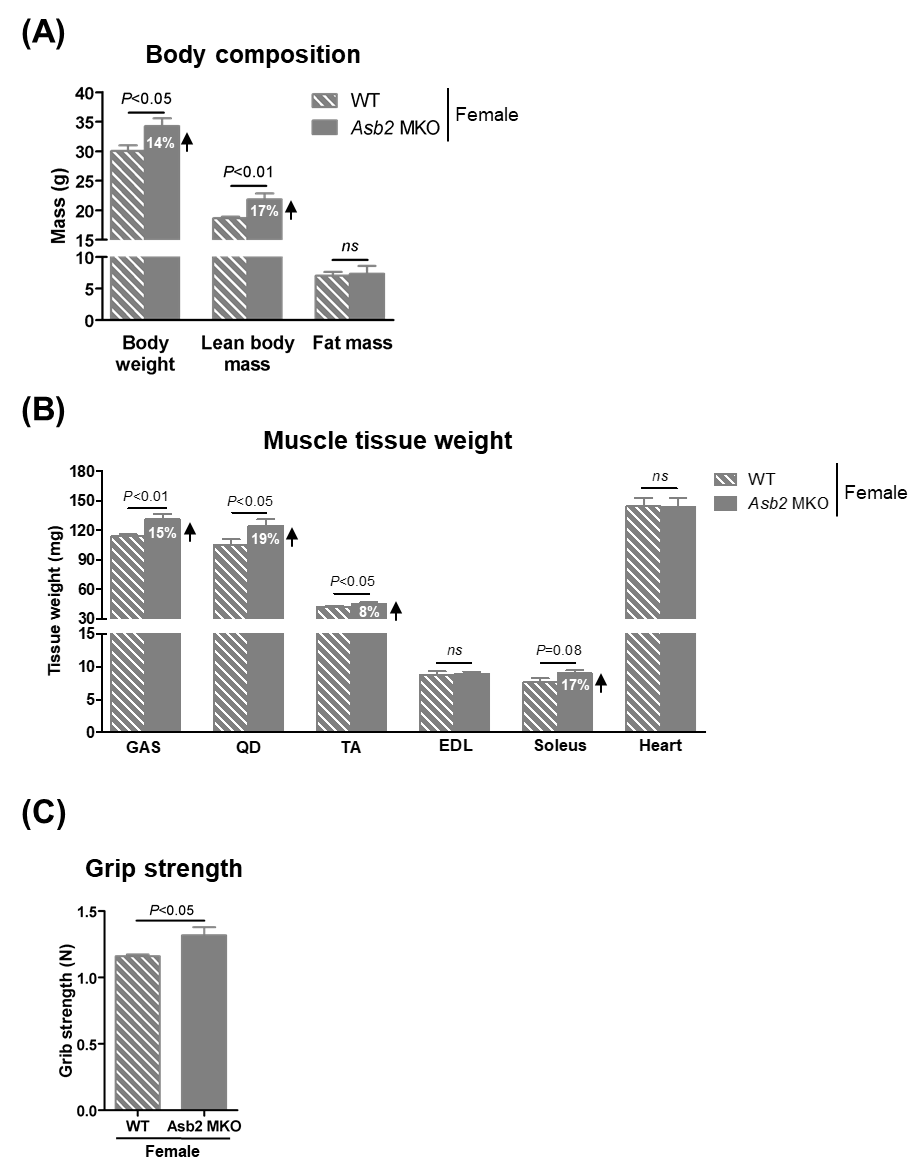
**

**Supplementary Figure S2. Deletion of the skeletal muscle-specific *Asb2* gene increased skeletal muscle tissue weight and grip strength regardless of sex**

(A) Body composition of aged 18-month-old WT and *Asb2* MKO female mice measured before fasting (n = 6~7 each). (B) Skeletal muscle and heart tissue weights of aged 18-month-old WT and *Asb2* MKO female mice fasted overnight prior to sacrifice (n = 6~7 each). (C) Grip strength of 18-month-old WT and *Asb2* MKO female mice fed a regular chow diet (n = 6~7 each). Data are represented as means ± standard error of the mean (SEM). Statistical significance was determined using Student’s *t* test. Not significant, ns; Gastrocnemius, GAS; Quadriceps, QD; Tibialis anterior, TA; Extensor digitorum longus, EDL.


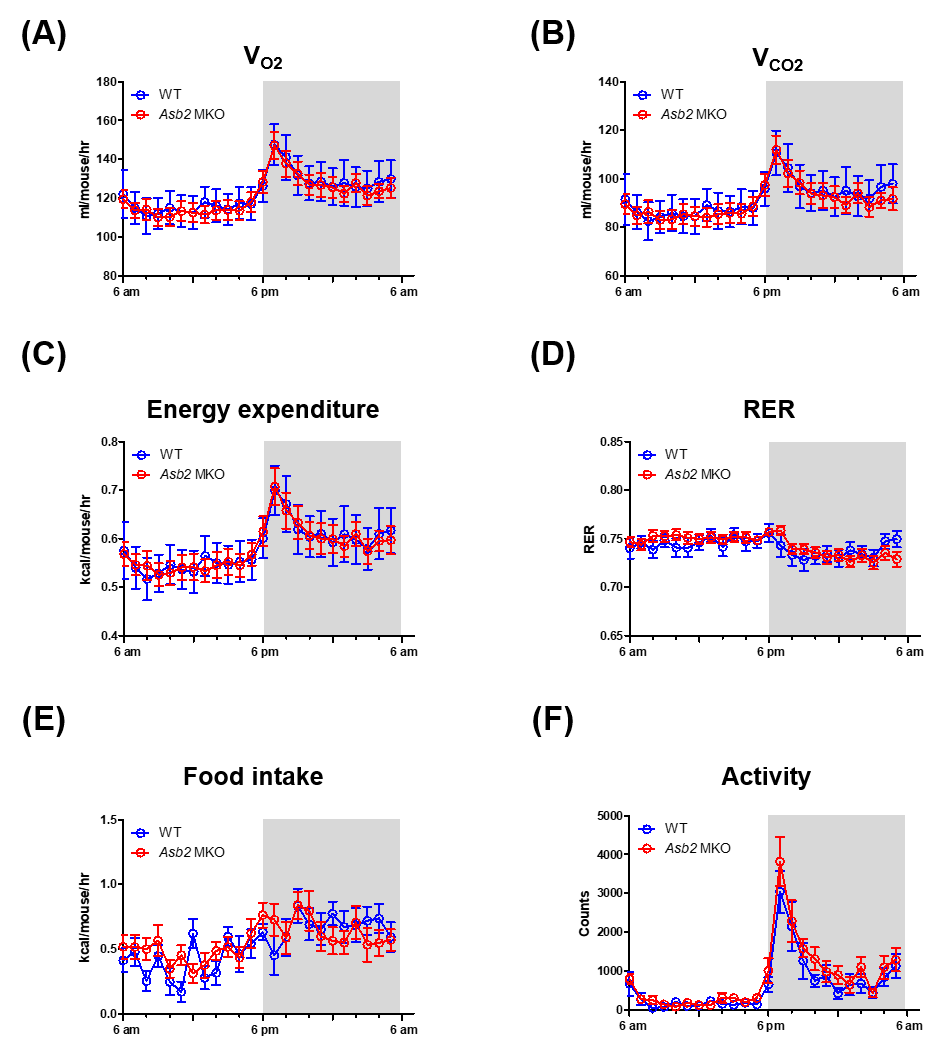


**Supplementary Figure S3. Energy expenditure of skeletal muscle-specific *Asb2* knockout mice under high-fat diet conditions**

Whole-body energy balance in 6-month-old WT and *Asb2* MKO male mice fed a high-fat diet (HFD) for 4 weeks. (A) Oxygen consumption (V_O2_), (B) carbon dioxide production (V_CO2_), (C) energy expenditure, (D) respiratory exchange ratio (RER), (E) food intake, and (F) activity were measured in mice housed in individual metabolic cages for 48 hours (n = 10 each). Data are represented as means ± standard error of the mean (SEM) and expressed per mouse.


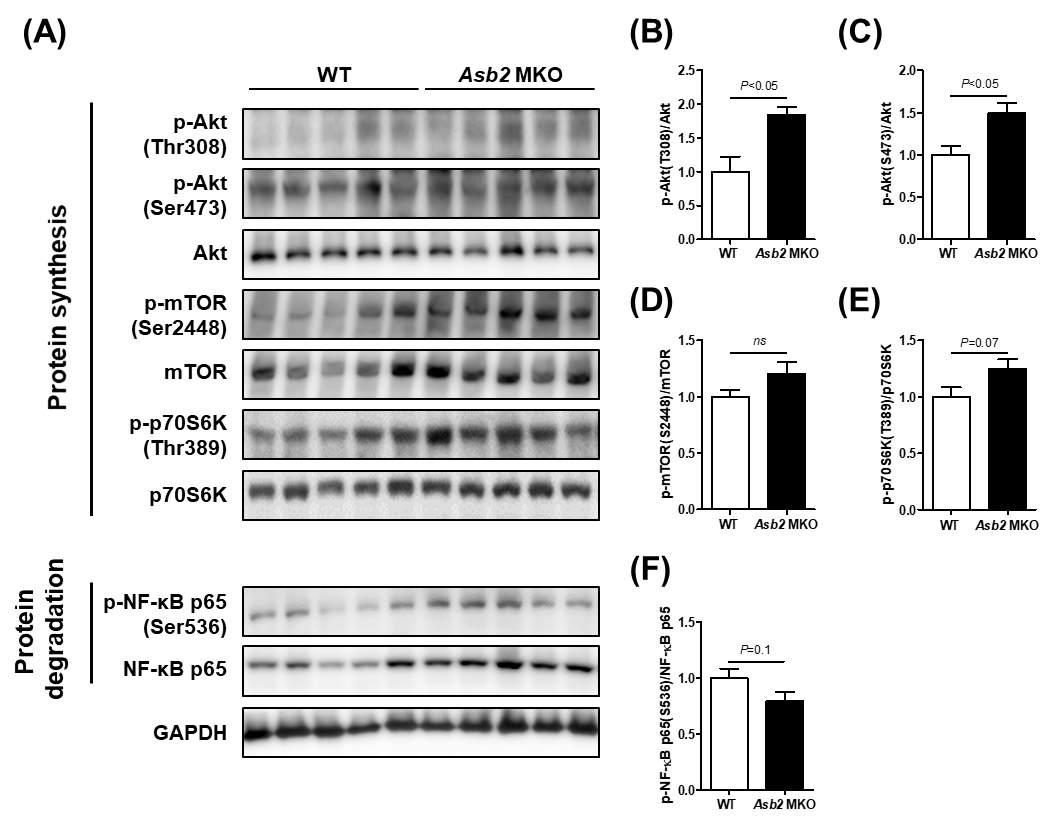


**Supplementary Figure S4.** **Skeletal muscle-specific *Asb2* knockout mice exhibited a tendency toward increased protein synthesis and decreased protein degradation signaling in gastrocnemius muscle.**

(A-F) Representative immunoblots of protein synthesis and protein degradation signaling in the gastrocnemius (GAS) muscle of 6-month-old WT and *Asb2* MKO male mice fasted overnight prior to sacrifice. The p-Akt (Thr308, Ser473), p-mTOR (Ser2448), p-p70S6K (Thr389), p-NF-κB P65 (Ser536) levels were analyzed by Western blotting, and quantified (n = 5 each). Data are represented as means ± standard error of the mean (SEM). Statistical significance was determined by Student’s *t* test. Not significant, ns; Ankyrin repeat and suppressor of cytokine signaling box protein 2, ASB2; Gastrocnemius, GAS; Mammalian target of rapamycin, mTOR; p70 ribosomal S6 kinase, p70S6K; Nuclear factor-κB, NF-κB; Glyceraldehyde-3-phosphate dehydrogenase, GAPDH.

**
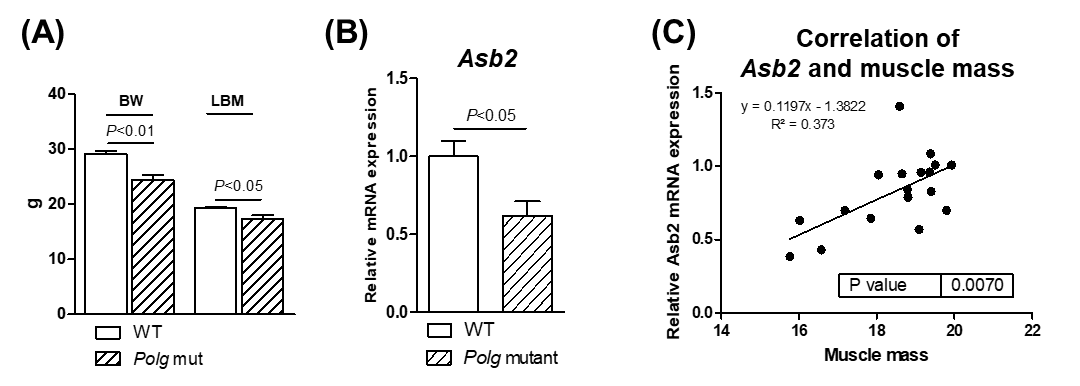
**

**Supplementary Figure S5. *Asb2* expression is associated with myopathy**

(A) Body weight and lean body mass of 6-month-old WT and mitochondrial polymerase γ mutant mice (*Polg* mut) male mice measured before fasting (n = 6 each). (B) Ankyrin repeat and suppressor of cytokine signaling box protein (*Asb2*) mRNA expression in the quadriceps muscle obtained from 6-month-old WT and *Polg* mut male mice fasted overnight prior to sacrifice (n = 6 each). (C) Correlation of *Asb2* mRNA levels and lean body mass in 6-month-old WT and *Polg* mut male mice (n = 18). Data are represented as means ± standard error of the mean (SEM). Statistical significance was determined using Student’s *t* test. Mitochondrial polymerase γ, *Polg*; Body weight, BW; Lean body mass, LBM; Ankyrin repeat and suppressor of cytokine signaling box protein 2, *Asb2*.
